# Calcium phosphate-based anti-infective bone cements: recent trends and future perspectives

**DOI:** 10.3389/fphar.2025.1522225

**Published:** 2025-02-26

**Authors:** Xiang Liu, Chaoli Wang, Han Wang, Guoliang Wang, Yong Zhang, Yunfei Zhang

**Affiliations:** ^1^ Department of Orthopaedics, Second Affiliated Hospital, Air Force Medical University, Xi’an, China; ^2^ Department of Pharmacy, Air Force Medical University, Xi’an, China

**Keywords:** calcium phosphate cement, resistance to antibiotics, bone infection, antibacterial agents, multifunctional bone cement

## Abstract

Bone infection remains a challenging condition to fully eradicate due to its intricate nature. Traditional treatment strategies, involving long-term and high-dose systemic antibiotic administration, often encounter difficulties in achieving therapeutic drug concentrations locally and may lead to antibiotic resistance. Bone cement, serving as a local drug delivery matrix, has emerged as an effective anti-infective approach validated in clinical settings. Calcium phosphate cements (CPCs) have garnered widespread attention and application in the local management of bone infections due to their injectable properties, biocompatibility, and degradability. The interconnected porous structure of calcium phosphate particles, not only promotes osteoconductivity and osteoinductivity, but also serves as an ideal carrier for antibacterial agents. Various antimicrobial agents, including polymeric compounds, antibiotics, antimicrobial peptides, therapeutic inorganic ions (TIIs) (and their nanoparticles), graphene, and iodine, have been integrated into CPC matrices in numerous studies aimed at treating bone infections in diverse applications such as defect filling, preparation of metal implant surface coatings, and coating of implant surfaces. Additionally, for bone defects and nonunions resulting from chronic bone infections, the utilization of calcium phosphate-calcium sulfate composite multifunctional cement loaded with antibacterial agents serves to efficiently deal with infection, stimulate new bone formation, and attain an optimal degradation rate of the bone cement matrix. This review briefly delves into various antibacterial strategies based on calcium phosphate cement for the prevention and treatment of bone infections, while also discussing the application of calcium phosphate-calcium sulfate composites in the development of multifunctional bone cement against bone infections.

## 1 Introduction

Bone infection, an inflammatory disease resulting from pyogenic bacterial infection, leads to osteolysis and necrosis. Its primary causes include trauma, orthopedic surgeries, joint replacements, and the dissemination of diabetic foot infection (DFI) lesions. Hematogenous diseases have also been implicated as a source of bone infection (Zhong et al., 2023; Kavanagh et al., 2018; Senneville et al., 2020). This condition is associated with high recurrence and disability rates, prolonged hospital stays, and a significant economic burden for patients (Kavanagh et al., 2018; Vallet-Regí et al., 2020; Schade et al., 2021). *Staphylococcus aureus* (*S. aureus*) infection is particularly prevalent, with approximately 30% of cases involving Methicillin-resistant *S. aureus* (MRSA) (Zhong et al., 2023; Nandi et al., 2016). The treatment of bone infection poses a significant challenge for both patients and orthopedic surgeons (Zhong et al., 2023; Geurts et al., 2021; McNally et al., 2020).

### 1.1 Analysis of the challenges associated with the treatment of bone infections

**FIGURE 1:**
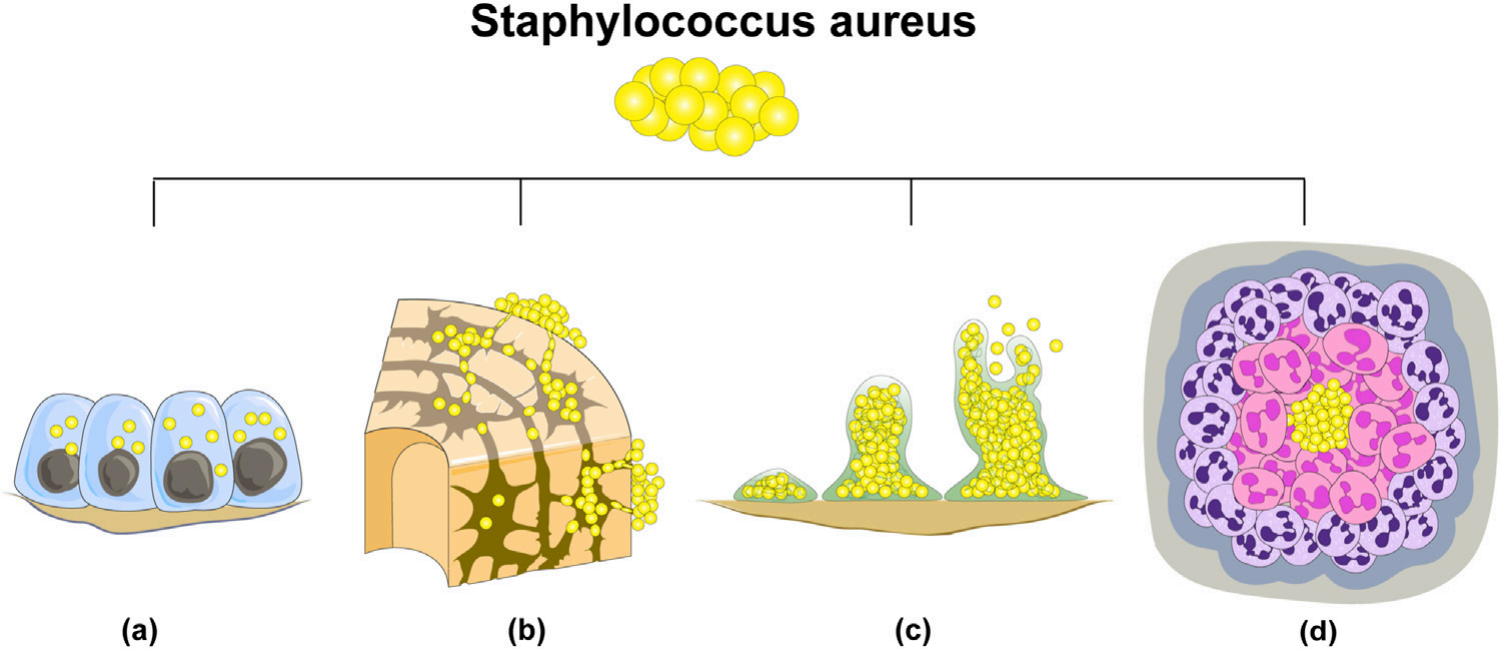
There are four principal reasons underlying the difficulty in eradicating *S. aureus* in bone infections: **(A)** intracellular bacterial colonization; **(B)** invasion of osteocyte lacuno-canicular network (OLCN); **(C)** biofilm formation; **(D)** abscess formation.

*S. aureus* stands as the foremost etiological agent responsible for bone infections, exhibiting the highest pathogenicity among microbial pathogens. The genesis of *S. aureus* infection is attributed to the emergence of drug-resistant bacterial strains and their evasion of host immune surveillance. The recalcitrant nature of osteomyelitis instigated by *S. aureus* primarily arises from intracellular infection within the host, invasion of osteocyte lacuno-canicular network (OLCN), biofilm formation, and the development of staphylococcal abscesses (Masters et al., 2022). (Figure 1) Concurrently, diabetic foot osteomyelitis (DFO) and soft tissue infection, recognized as significant complications of diabetes, pose formidable treatment challenges and impart a considerable burden on both public health systems and individual patients (Leone et al., 2020; Senneville et al., 2024; Rubitschung et al., 2021).

#### 1.1.1 Intracellular bacterial colonization

Extensive research has demonstrated that *S. aureus* is capable of persisting within a diverse range of cellular environments, including macrophages, osteoblasts, osteoclasts, osteocytes, and fibroblasts, for prolonged durations (Masters et al., 2022). Macrophages harboring intracellular bacterial invasion are referred to as “Trojan horse” macrophages, facilitating the accumulation of small bacterial colony variants (Garzoni and Kelley, 2011). This intracellular residency of *S. aureus* accelerates osteoblast apoptosis and further augments osteoclast activity. Additionally, when pathogenic bacteria colonize bone cells, they trigger the secretion of inflammatory factors that induce osteoclasts, ultimately leading to pathological bone loss (Tong et al., 2022; Yoshimoto et al., 2022). Furthermore, *S. aureus* colonization within osteoblasts promotes their differentiation and maturation into osteocytes, serving as a vehicle for long-term immune evasion within the host (Alder et al., 2020; Watkins and Unnikrishnan, 2020).

#### 1.1.2 OLCN invasion

The invasion of OLCN by *S. aureus* represents a novel mechanism of bacterial persistence and immune evasion in chronic osteomyelitis (Masters et al., 2021). Traditionally, *S. aureus* was considered a non-motile bacterium. However, recent advancements in research have established that *S. aureus* is capable of invading and residing within OLCN for extended durations (Yu et al., 2020). Notably, it has been demonstrated that the bacterium can successfully traverse the narrow spaces within the tubules by altering its shape (Jensen et al., 2023). *In vitro* investigations have revealed that the cell wall transpeptidase-penicillin-binding protein 4 (PBP4) and surface adhesin-*S. aureus* surface protein C (SasC) play pivotal roles in *S. aureus*’s ability to deform and replicate during its traversal through the tubules (Masters et al., 2021).

#### 1.1.3 Biofilm formation

The intricate mechanism underlying biofilm formation during osteomyelitis has garnered significant attention in recent research. Biofilms, particularly those formed by *S. aureus* play a vital role. in persistent infections. Firstly, the biofilm matrix, composed of extracellular polymeric substances (EPS) including polysaccharides, proteins, and extracellular DNA secreted by bacteria during growth, serves as a protective barrier. This matrix not only hinders the diffusion of antibiotics to bacteria within the biofilm but also inhibits the penetration of immune cells, thereby contributing to bacterial persistence (Masters et al., 2022; Schilcher and Horswill, 2020). Secondly, the interaction between EPS and bacterial aggregates confers cohesion and viscoelasticity to the biofilm, enabling bacteria to adhere firmly to both biotic and abiotic surfaces and resist external mechanical forces (Peng et al., 2022). Thirdly, *S. aureus* exhibits altered metabolic phenotypes within biofilms, rendering it more resilient against the effects of antibiotics (Muthukrishnan et al., 2019). Additionally, the accessory gene regulator (Agr) quorum sensing system serves as a crucial regulator of *S. aureus* biofilm formation, which is intricately linked to imbalances in bone homeostasis and disease progression (Masters et al., 2022; Butrico and Cassat, 2020).

#### 1.1.4 Staphylococcal abscess formation

Abscess formation serves as an additional mechanism for the long-term survival and immune evasion of *S. aureus*. Initially, *S. aureus* overcomes the host’s innate immune defense mechanisms by expressing microbial surface components recognizing adhesive matrix molecules (MSCRAMMs). As *S. aureus* cells accumulate and establish colonies, a significant influx of neutrophils occurs in the affected area. These neutrophils undergo necrosis due to the effects of *S. aureus*, inadvertently creating a “protective barrier” that hinders the ability of newly recruited host immune cells to eliminate the bacteria within the abscess. Eventually, as the abscess ruptures, the bacteria disseminate to new anatomic locations, perpetuating the infection process (Masters et al., 2022; Muthukrishnan et al., 2019).

Furthermore, as inflammation ensues, the delicate balance between osteoblasts and osteoclasts within the local bone tissue is disrupted. Osteoclasts, the sole cell type responsible for bone resorption, exhibit excessive differentiation and proliferation, which are intricately linked to bone loss and ultimately give rise to infectious bone defects. *S. aureus* can directly bind to osteoclasts and promote bone resorption through its own virulence factors, such as Staphylococcal protein A (SpA), peptidoglycan, and lipoproteins. Additionally, it can indirectly stimulate osteoclasts to increase bone resorption by upregulating the synthesis of macrophage colony-stimulating factor (M-CSF) and receptor activator of nuclear factor-κB ligand (RANKL) in osteoblasts. Notably, the activated immune system generates a copious amount of proinflammatory cytokines, which further promote osteoclast differentiation and exacerbate bone resorption (Tong et al., 2022; Mendoza et al., 2016; Kassem and Lindholm, 2016; Udagawa et al., 2021).

#### 1.1.5 DFI

The involvement of bone as a secondary complication of DFIs is particularly severe, especially in cases of the most advanced soft tissue DFIs. Therefore, a high degree of vigilance is essential in the occurrence of DFO (Senneville et al., 2020).

DFIs are defined as infections involving soft tissue or bone located anywhere below the ankle in diabetic patients. These infections frequently originate from diabetic foot ulcers (DFUs) and are closely correlated with the eventual risk of lower limb amputation in affected patients (Noor et al., 2017; Mohseni et al., 2019). Multiple pathological factors elevate the risk of foot infections in individuals with diabetes, including neuropathy, vascular insufficiency, immune dysfunction, and alterations in foot biomechanics, all of which are contributing elements to the development of DFIs (Rubitschung et al., 2021; Noor et al., 2017). Neurological complications in diabetic patients encompass motor, sensory, and autonomic dysfunction. Motor neuropathy results in atrophy of foot muscles and associated deformities, leading to traumatic injuries. Impaired sensory function often leads to the body overlooking damage to the lower limbs, fostering a vicious cycle of injury. Autonomic neuropathy disrupts normal blood flow to the soles of the feet. When accompanied by a loss of sweat and sebaceous gland function, the skin becomes dry and keratinized, rendering it more susceptible to rupture and serving as a portal for initial infection (Pitocco et al., 2019). Concurrently, the presence of peripheral artery disease exacerbates tissue ischemia, impairs wound healing processes, and fosters an environment conducive to infection (Armstrong et al., 2023). Hyperglycemia-induced decreases in host defense capacity encompass deficiencies in neutrophil function, alterations in macrophage morphology, elevations in proinflammatory cytokines, and impairments in diabetic polymorphonuclear cell functions, including chemotaxis, phagocytosis, and bactericidal activity. Additionally, locally elevated blood glucose levels serve as an optimal medium for enhancing the virulence of pathogenic bacteria (Baroni and Russo, 2014).

Given that DFIs are the culmination of multiple factors, their treatment necessitates a multidisciplinary and coordinated comprehensive approach. Effective antibacterial interventions and necessary surgical procedures are pivotal. Local antimicrobial therapy and strategies to combat local drug resistance, encompassing antibiotic-impregnated biomaterials, novel antimicrobial peptides, nanomedicine, and photodynamic therapy, represent key considerations in the management of DFIs (Maity et al., 2024). Clinicians are continually focused on addressing how to facilitate the healing of locally damaged tissue, which involves revascularization, while rigorously managing blood glucose levels. Local growth factors, shockwave therapy, negative pressure wound therapy, and stem cell therapy have emerged as promising avenues for accelerating wound healing and reducing the incidence of DFUs as well as the amputation rate (Raja et al., 2023).

### 1.2 Recent advances in local treatment of bone infection

Conventional treatment strategies for bone infection have historically encompassed surgical debridement and long-term, high-dose systemic antibiotic therapy. However, these approaches exhibit notable limitations. Firstly, the attainment of effective antibiotic concentrations at the lesion site is challenging due to bone loss, sequestrum formation, and soft tissue insufficiency (Zhong et al., 2023). Secondly, bone loss at the osteomyelitis lesion and surgical debridement-induced bone defects pose significant challenges for natural healing, particularly when exceeding a certain threshold, significantly compromising patient recovery and quality of life (Yoshimoto et al., 2022; Migliorini et al., 2021). Furthermore, since the introduction of antibiotics in osteomyelitis treatment in the 1940s, the emergence of bacterial resistance has garnered increasing attention from orthopedic surgeons, posing a significant obstacle in the therapeutic process (Zhong et al., 2023; Wang X. et al., 2023).

As an effective and feasible alternative strategy to solve the above shortcomings, local treatment has been successfully used in clinical practice (Zhong et al., 2023). The utilization of multifunctional antibacterial materials in clinical practice has successfully demonstrated their therapeutic potential in achieving slow-release of high-dose local antibacterial agents, effectively eradicating bacteria, and promoting angiogenesis and bone formation (Wang X. et al., 2023; Cobb and McCabe, 2020). Notably, the integration of antibacterial drugs with bone cement in a local drug delivery system has proven effective in both preventing and treating bone infections, while simultaneously enhancing osseointegration efficiency of bone cement implants (Alegrete et al., 2023; Boyle et al., 2019; Wu et al., 2021). Currently, three types of bone cement are commonly employed in clinical settings for the local treatment of bone infections: poly (methyl methacrylate)(PMMA) cements, CPCs, and calcium sulfate cements (CSCs). Each of these cements exhibits distinct characteristics that contribute to their respective therapeutic outcomes (Quan et al., 2023).

## 2 Essential characteristics of CPCs for medical applications

CPCs are defined as a combination of one or multiple calcium phosphate powders or particles. Upon mixing with the corresponding liquid phase, they transform into a paste capable of solidifying and hardening within the bone defect site, ultimately forming a scaffold (Ginebra et al., 2012). The common CPCs encompass tricalcium phosphate (TCP), tetracalcium phosphate (TTCP), octacalcium phosphate (OCP), hydroxyapatite (HA), and calcium-deficient hydroxyapatite (CDHA), among others. Unlike acrylic bone cements, which are hardened by polymerization reactions, CPCs are the result of a dissolution and precipitation process and fall into two main categories: precipitated apatite cement and brushite cement. In the context of apatite cement formation, CDHA is formed when a single-composition calcium phosphate compound undergoes hydrolysis without altering the Ca/P ratio. Conversely, precipitated HA results from an acid-base reaction yielding multiple calcium phosphate compounds. Brushite cement is primarily obtained through the acid-base reaction involving more than one type of calcium phosphate (Ginebra et al., 2012; Vezenkova and Locs, 2022). Under physiological conditions, brushite cement exhibits more rapid dissolution rates in comparison to apatite cement (Ginebra et al., 2012; Apelt et al., 2004). CPCs possess injectable properties and their composition closely resembles the chemical structure of bone mineral. HA, a bioactive inorganic ceramic with a chemical and crystal structure resembling that of natural bone apatite [Ca_10_(PO_4_)_6_(OH)_2_], exhibits excellent biological properties (Fang et al., 2006). When compared to PMMA cements, CPCs exhibit superior biocompatibility and bioabsorbability. Their degradation products provide essential calcium and phosphate ions at the implantation site, vital for local mineralization. Furthermore, CPCs demonstrate greater osteoconductivity and osteoinductivity than CSCs, thereby facilitating new bone formation (Ginebra et al., 2012; Vezenkova and Locs, 2022).

**FIGURE 2:**
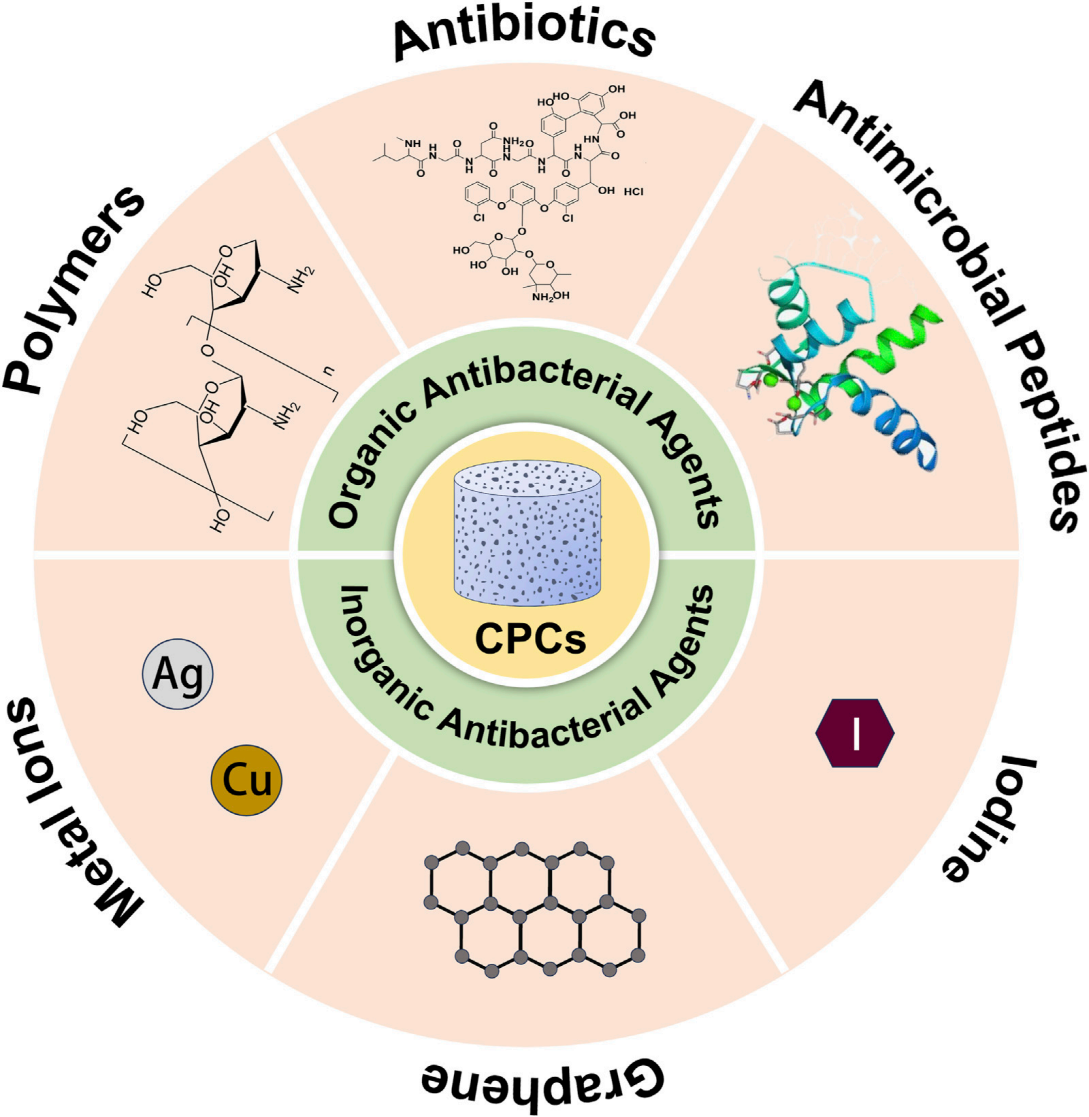
Antibacterial agents loaded in calcium phosphate bone cement.

The inherent interconnected macropores (pore size> 100 μm) and micropores (pore size <100 μm) of CPCs not only surpass CSCs in osteoconductivity and osteoinduction, thereby promoting new bone formation (Ginebra et al., 2012; Vezenkova and Locs, 2022), but also expand their specific surface area for the adsorption of bioactive substances. These substances, including drugs, bioactive molecules, and metal or non-metal ions, further enhance the functionality of CPCs (Ginebra et al., 2012; Vezenkova and Locs, 2022; Grosfeld et al., 2016; Kost et al., 2023) (Figure 2)

Furthermore, CPCs possess the ability to harden at normal or room temperature, permitting bioactive substances to penetrate their inherent pores without compromising their activity (Ginebra et al., 2012; Vezenkova and Locs, 2022; Rh Owen et al., 2018). Consequently, CPCs exhibit exceptional biocompatibility *in vivo* and serves as an optimal drug delivery system for the locally controlled release of antibacterial agents.

## 3 CPC-based bone filler for the treatment of bone infection

From a clinical perspective, selecting appropriate CPC application strategies tailored to patients’ individual conditions is pivotal for disease treatment. The appropriate form of CPC implantation is closely associated with various patient factors, including whether the bone defect is located in a weight-bearing area, its size, and whether it is a complex defect. Furthermore, certain intrinsic properties of CPC also influence the choice of its final application form, encompassing porosity, mechanical strength, injectability, solidification reaction and time, cohesion, biodegradability, and the inclusion of additives. These factors are interdependent and collectively determine the ultimate characteristics of CPC in practical applications (Ginebra et al., 2012; Vezenkova and Locs, 2022; Mucalo, 2019). In cases where bone infection leads to significant defects, the Masquelet technique is initially employed for membrane induction, followed by autologous bone transplantation. To address the defects, larger porous particles of CPC, which have been pre-solidified and sterilized prior to surgery, can be utilized as a replacement (Andrzejowski et al., 2020; Gupta et al., 2019). It is noteworthy that CPC particles exhibit brittleness and possess limited mechanical strength, with pores playing a crucial role in this context (Baudín et al., 2019). This limits application to weight-bearing sites. Additional interventions, such as internal fixation, are necessary to augment the mechanical strength of the affected region. Ideally, the mechanical properties of the implant should closely approximate those of the surrounding bone tissue (Vezenkova and Locs, 2022). On the other hand, in cases where the bone defect is small or irregularly shaped, to prevent cavity residuals, the use of paste-form CPC with high injectability is recommended for cavity filling (Demir-Oğuz et al., 2023). The injectability of the material is intimately associated with the ratio of solid to liquid components, as well as the particle size distribution of the material (Vezenkova and Locs, 2022). Luo et al. (2018) developed a pre-mixed acid cement formulated from monocalcium phosphate monohydrate (MCPM) paste and β-TCP paste, specifically tailored for the rapid and minimally invasive filling of bone defects. This approach is effective in reducing procedure time and minimizing the risk of contamination.

For the drug loading of CPCs, two primary methods can be employed (Parent et al., 2017): direct loading and indirect loading. Direct loading involves the mixing of solid or liquid antibacterial drugs directly with the bone cement prior to administration (Yu et al., 2010). During surgical intervention, when CPC powder is intended for mixing with the corresponding curing liquid, the therapeutic agent is generally incorporated directly within the bone cement compound. While this approach enables CPCs hardening at low temperatures, thereby preventing drug denaturation or inactivation that might occur at higher temperatures as compared to PMMA (Ginebra et al., 2012; Vezenkova and Locs, 2022; Rh Owen et al., 2018), it is not without its limitations. These include a lack of standardization in the production process, inhomogeneous drug distribution within the bone cement matrix, restricted drug quantities to maintain mechanical strength, and challenges in achieving an optimal *in vitro* release profile for macromolecular drugs (Mouriño and Boccaccini, 2010; Chen L. et al., 2024). Indirect loading, on the other hand, involves impregnating a calcium phosphate carrier in a drug solution or suspension, followed by separation of the impregnated carrier from the liquid phase and evaporation of the solvent to obtain a dry drug-loaded carrier (Wang et al., 2022). This method is particularly suitable when the micro three-dimensional structure of CPCs is crucial for exerting its therapeutic effects and when the fragility of the material is a concern (Parent et al., 2017). When selecting pre-mixed bone cement prior to surgery, antibiotics can be incorporated via dipping to enhance their antibacterial efficacy. However, the limitation of a shortened shelf life associated with this method cannot be overlooked (Luo et al., 2018).

The regulation of release kinetics from a drug carrier is pivotal in maintaining the therapeutic window, defined as the concentration range between the minimal inhibitory concentration for bacteria and the minimal toxic concentration for humans, for the administered drug (McInnes et al., 2016; Wu et al., 2024). A research team has demonstrated the efficacy of utilizing calcium phosphate-loaded antibiotics for the localized treatment of bone infections. Notably, the serum concentration of the antibiotic in patients remains low, staying below the manufacturer-recommended safety limits (Sasaki et al., 2005). For a constant quantity of a given drug, when blended directly with CPC matrix components, drug release is primarily governed by matrix diffusion, with the concentration and duration of release exhibiting a positive correlation with the drug dosage. Conversely, when the drug is adsorbed onto the substrate surface through incubation, the release mechanism becomes anomalous, displaying an increase in release as the drug loading augments (Fosca et al., 2022).

The rate of drug release from the carrier is influenced by various factors, encompassing not only the method of drug loading but also characteristics of the bone cement matrix, drug type, drug loading quantity or concentration, and the utilization of additives (Parent et al., 2017; Fosca et al., 2022). Specifically, the porosity, pore size distribution, specific surface area, and crystallinity of the bone cement matrix all play a significant role. Furthermore, the addition of polymer additives to the CPC matrix represents a relatively straightforward approach to regulate drug release kinetics.

### 3.1 Organic antibacterial substances loaded with CPCs

#### 3.1.1 Polymer-doped CPCs

By incorporating biodegradable polymers, such as chitosan (CS), mannitol, hyaluronic acid, alginate, gelatin, collagen, hydroxypropyl methyl cellulose (HPMC), and poly (lactic-co-glycolic acid) (PLGA), into the drug-loaded matrix under specific conditions, it is possible to mitigate the initial burst release of drugs from the carriers. This modulation results in a more sustained and controlled drug release profile from the cement matrix (Vezenkova and Locs, 2022; Fosca et al., 2022; Mistry et al., 2016; Xu et al., 2017; Dorozhkin, 2019), thereby effectively fulfilling the objective of preventing and treating bone infection.

The influence of additives on the drug release rate within CPC manifests firstly through alterations in the drug’s affinity for the matrix, where a negative correlation exists between affinity and release rate. Secondly, the intrinsic properties of the additives themselves can modulate the rate of drug release. When polymers are employed as additives, they induce a spatial effect by swelling and filling the pores of the matrix, thereby inhibiting drug release. Subsequently, as the polymer undergoes degradation, the release rate augments due to the facilitated diffusion of drug molecules (Vezenkova and Locs, 2022; Fosca et al., 2022; Li et al., 2007; David Chen et al., 2011). When the local concentrations of antibiotics released by CPC remain subtherapeutic for an extended period following the initial burst release, the potential for bacterial resistance may arise (Thomes et al., 2002; Kendall et al., 1996). The incorporation of a polymer as an additive in the CPC matrix results in a reduction of the initial burst release of the drug and promotes a more sustained release profile of the drug over time (David Chen et al., 2011; Jin, 2015). This characteristic of the polymer is advantageous for modulating drug release within the therapeutic window in CPC, thereby mitigating the adverse effects of bacterial resistance and ensuring the antibacterial efficacy of the treated area within a safe concentration range. Furthermore, the rate of drug release in the CPC matrix can be controlled by adjusting the concentration of the polymer in the reaction solution (Tiğli et al., 2009).

Normal human bone comprises a complex combination of inorganic and organic materials. By incorporating polymeric organic polymers into CPC, we can better mimic the natural state of bone tissue, thereby enhancing the biocompatibility of CPC *in vivo*. Additionally, the inclusion of these polymers is advantageous in improving the injectability and mechanical strength of the bone cement matrix. Furthermore, it has been demonstrated that the antimicrobial properties of drug-loaded cement are also enhanced through the addition of polymers (Wu et al., 2021; Wu et al., 2020a; Bose et al., 2023).

CS is a polysaccharide compound derived from chitin through deacetylation (Bakshi et al., 2020). Its molecular structure harbors multiple functional groups, facilitating modifications and chemical reactions (Zhong et al., 2023). Apart from its biodegradability and biocompatibility, CS exhibits a strong affinity towards negatively charged bacterial membranes due to its cationic nature, thereby displaying moderate antibacterial activity (Tao et al., 2020; Negm et al., 2020). Yang et al. (2018) successfully fabricated a PLGA-HA scaffold grafted with CS using 3D printing technology. Both *in vitro* and animal experiments confirmed that the CS-PLGA-HA scaffold demonstrated remarkable antibacterial properties against *S. aureus*, along with bone conductivity, offering a novel approach to enhance the local therapeutic efficacy of CPCs in the treatment of infectious bone defects.

#### 3.1.2 Antibiotic-loaded CPCs

Antibiotics have long been extensively studied as a primary approach for preventing and treating bone infections. Surgical procedures such as wound contamination, fracture repair with or without internal fixation, joint prosthesis implantation, and spinal surgery can often lead to severe bone infections. Therefore, the successful osseointegration of CPCs during surgical procedures critically depends on the loading of antibacterial drugs, which effectively prevents bone infection at the surgical site (Ginebra et al., 2006). To prevent bone infection, it is essential that the antibiotics released from the bone cement carrier rapidly reach the minimum inhibitory concentration (MIC) of the corresponding pathogenic bacteria, while avoiding prolonged exposure to sub-inhibitory concentrations that may promote bacterial resistance. For the treatment of bone infection, it is necessary to achieve local long-term antibiotic release (Ginebra et al., 2012).

Currently, the antibiotics loaded into CPCs for the treatment of bone infections are primarily derived from a diverse range of antimicrobial agents. These include, but are not limited to, aminoglycosides such as gentamicin sulfate, tobramycin, and amikacin; β-lactams like meropenem and hydroxy benzylpenicillin; glycopeptides such as vancomycin and teicoplanin; quinolones like moxifloxacin and ciprofloxacin; and tetracyclines, such as doxycycline. These antibiotics, used singly or in combination, have demonstrated effectiveness in the prevention and treatment of bone infections (Ginebra et al., 2012; Mistry et al., 2016; Ribeiro et al., 2022; Nasser et al., 2022; Mabroum et al., 2023; Radwan et al., 2021; Liu et al., 2023; Mistry et al., 2022; Pradeep et al., 2023; Bhanwala and Kasaragod, 2022; Kumar Dewangan et al., 2023) (Table 1)

**TABLE 1:** Antibiotics loaded into calcium phosphate bone cement.

Antibiotic type	Antibiotic(s)	CPC Ingredient(s)	Antibacterial efficiency	References
Aminoglycosides	Gentamicin	On the basis of the commercially available CPC (Neocement^®^), CS and HPMC were added to enhance its performance and ensure sustained release of gentamicin	The formula containing 42% Liquid phase + HPMC + 1.87% wt gentamicin was identified as the optimal formulation, exhibiting desirable coagulation and mechanical properties, with an injectability of approximately 87% (compared to the original Neocement at 31%). This formulation ensures local release of gentamicin for 14 days at concentrations above antibacterial levels, demonstrating excellent antibacterial activity against *S. epidermidis* and moderate activity against *S. aureus*	Ribeiro et al. (2022)
β-lactams	Amoxicillin (AMX)	Citrate-modified mesoporous hydroxyapatite nanocarrier (Ctr-mpHANCs)	*In vitro*: AMX @ Ctr-mpHANs significantly reduced the growth of *S. aureus*, *E. coli*, and *P. aeruginosa* compared to Ctr-mpHANs	Nasser et al. (2022)
Quinolones	Ciprofloxacin	A reactive mixture containing 25% wt of bioactive glass (46S6) powder, along with an equimolar blend of calcium carbonate and dicalcium phosphate dihydrate, was prepared. Subsequently, these reactive powders were combined with a gel consisting of sodium alginate dissolved in a 0.25 M disodium hydrogen phosphate solution, maintaining an Liquid/Powder ratio of 0.7	*In vitro*: the cement composite loaded with ciprofloxacin exhibited excellent antibacterial activity against *S. aureus* and *E. coli*	Mabroum et al. (2023)
	Moxifloxacin	Biodegradable composite scaffolds of poly-lactide-co-ε-caprolactone/calcium phosphate (calcium phosphate: commercial-β-TCP, commercial-HA, commercial-dicalcium hydrogen phosphate)	The composite calcium phosphate scaffold effectively reduces bacterial load, inflammation, and sequestrum formation in the local lesions of an animal model of chronic osteomyelitis caused by *S. aureus*. As a result, it represents a promising candidate material for further clinical trials in the treatment of chronic osteomyelitis	Radwan et al. (2021)
Tetracycline class	Doxycycline	α-TCP (CAM Bioceramics B.V.) powders contained: 40 wt% PLGA, 1.5 wt% carboxymethyl cellulose (CMC), or 39.4 wt% PLGA plus 1.5 wt% CMC	*In vitro*: doxycycline released from the bone cement retained its antibacterial activity against *S. aureus*. Animal implantation models: a rapid reduction in the number of *S. aureus* bacteria on the surface of CPC and in surrounding tissues following implantation	Liu et al. (2023)
Glycopeptides (with other antibiotics)	Vancomycin Tobramycin	50 wt% HA+50 wt% β-TCP+α-CSH. After drug loading, BCP particles were encapsulated with PLGA for sustained release	Utilizing a rabbit tibial osteomyelitis model (MRSA), the superiority of composite bone cement in controlling infection rates and promoting bone healing was demonstrated compared to PMMA bone cement and parenteral therapy alone	Mistry et al. (2016)
			In the treatment of patients with chronic osteomyelitis (MRSA), composite bone cement exhibits superior performance in terms of cellular compatibility, hemostatic activity, infection control effectiveness, and bone regeneration compared to PMMA bone cement and parenteral therapy	Mistry et al. (2022)
	Vancomycin Amikacin	TTCP + Phosphoserine (PS) (The mass ratio: 10:6)	*In vitro*: antibacterial bone cement exerted inhibitory effects on the formation of biofilms by *E. coli*, *K. pneumoniae*, and *S. aureus*, and also suppressed the growth of these bacteria when cocultured. *In vivo*: the application of bone cement to rats with sternal infections caused by *S. aureus* or *E. coli* significantly inhibited bacterial activity	Pradeep et al. (2023)
	Vancomycin Meropenem	HA	A case report about Single step treatment of frontal sinus osteomyelitis using bone cement. During the follow-up period, no intracranial, nasal, or intraorbital complications were observed. Additionally, no recurrence or residual disease was detected at the conclusion of the 6-month period	Bhanwala and Kasaragod (2022)
	Vancomycin Gentamicin Meropenem Rifampicin	CDHA	*In vitro* experiments have demonstrated that eggshell-derived apatite bone cement is a more suitable injectable bone substitute for anterior use compared to injection-molded synthetic bone cement, effectively avoiding postoperative implant-related and other types of bone infections	Kumar Dewangan et al. (2023)
	Vancomycin	α-TCP based CPC modification with vancomycin loaded poly (lactic acid) (PLA) microcapsules	CPC modification with vancomycin loaded PLA microcapsules decreased the initial burst release of drug down to 7.7% ± 0.6%, while only 30.4% ± 1.3% of drug was transferred into the dissolution medium within 43days, compared to pure vancomycin loaded CPC, where 100% drug release was observed already after 12days	Loca et al. (2015)
	Teicoplanin	An injectable drug delivery system based on poloxamer 407 hydrogel containing undoped Mg, Zn-doped β-TCP, and teicoplanin	The encapsulated teicoplanin showed a sustained release over the evaluated period, enough to trigger antibacterial properties against Gram-positive bacteria. Besides, the formulations were biocompatible and showed bone healing ability and osteogenic properties. Finally, *in vivo* studies confirmed that the proposed locally injected formulations yielded osteomyelitis treatment with superior outcomes than parenteral administration while promoting bone regeneration	Kai et al. (2024)
	Teicoplanin	CPC powder containing 3% teicoplanin	To assess the effectiveness of calcium phosphate as a delivery system of teicoplanin, MRSA osteomyelitis was induced in 36 rabbits. And calcium phosphate cement with 3% teicoplanin was implanted. Bacterial eradication signified a considerable decrease of the total histologic scores of osteomyelitis compared with controls, accompanied with newly growing host bone	Lazarettos et al. (2004)
	Teicoplanin	CPC (Biopex; Pentax, Tokyo, Japan)	A 71-year-old man developed skin ulceration with cranial osteomyelitis after bypass surgery for diffuse cerebral infarction and internal carotid artery obstruction. MRSA was detected on wound culture test. Cranioplasty with a combination of calcium phosphate bone cement impregnated with teicoplanin, and a titaniummesh sheet and scalp reconstruction were performed. As of 6 months after surgery, no infection has relapsed	Ogino et al. (2020)

*S. epidermidis*, *Staphylococcus* epidermidis; *S. aureus*, *Staphylococcus aureus*; *E. coli*, *Escherichia coli*; *P. aeruginosa*, *Pseudomonas aeruginosa*; MRSA, Methicillin-resistant *Staphylococcus aureus*; *K. pneumoniae*, *Klebsiella pneumoniae*.

However, the emergence of antibiotic-resistant bacteria, including those that are refractory to almost all antibiotics and the challenges associated with the removal of biofilms, has garnered increasing attention. Consequently, the development of effective non-antibiotic alternatives has become a significant research focus in recent years (Aslam et al., 2018; Nir-Paz et al., 2019; Kalelkar et al., 2022). Additionally, the inclusion of antibiotics in bone cement matrices has been shown to compromise their mechanical stability, further emphasizing the need for alternative antimicrobial strategies (Arciola et al., 2018).

#### 3.1.3 Antimicrobial peptide-loaded CPCs

In the context of the escalating problem of antibiotic resistance in pathogenic bacteria, particularly caused by multidrug-resistant pathogens and biofilm formation (Peng et al., 2022; Muthukrishnan et al., 2019; van Staden and Heunis, 2011), the urgent need to identify reliable alternatives to antibiotics has become paramount. This is due to the difficulties in discovering new antibiotics that can effectively address this challenge in the near future (Volejníková et al., 2019; Sinha and Shukla, 2019; Costa et al., 2021).

Antimicrobial peptides (AMPs) represent a class of basic active peptides that exhibit broad-spectrum antibacterial activity against both Gram-positive and Gram-negative bacteria. These peptides demonstrate high antimicrobial efficacy even at low concentrations, possess anti-biofilm activity, and exert immunomodulatory effects (Costa et al., 2021; Melicherčík et al., 2018; Yazici et al., 2019; Luo and Song, 2021). Given that AMPs target multiple components within the plasma membrane and cytosol of bacteria, the likelihood of them inducing pathogen resistance is extremely low (Costa et al., 2021; Van Staden and Dicks, 2012). Currently, several AMPs have been approved by the U.S. Food and Drug Administration (FDA) for the treatment of severe bacterial infections, underscoring their potential as viable alternatives to traditional antibiotics (Dijksteel et al., 2021).

Notably, the application of drug-eluting coatings loaded with antimicrobial peptides onto porous calcium phosphate substrates represents a promising strategy for the prevention of orthopedic implant-related infections and the formation of biofilms on their surfaces:

Kazemzadeh-Narbat et al. (2010) selected the broad-spectrum AMP Tet213 and applied it to a titanium surface, creating coatings composed of microporous OCP as a drug carrier. These coatings, with a thickness of 7 μm and an AMP loading of 9 μg/cm^2^, exhibited antimicrobial activity *in vitro* against both *S. aureus* (Gram-positive) and *P. aeruginosa* (Gram-negative). These results indicate that calcium phosphate-Tet213 coatings could potentially serve as an effective solution for the prevention of infections associated with orthopedic implants. Furthermore, the team demonstrated significantly lower *in vitro* cytotoxicity (200 μg/mL) of AMP HHC36 compared to AMP Tet213 (50 μg/mL) in calcium phosphate-HHC36 coatings applied to a titanium surface. Additionally, these coatings exhibited antimicrobial activity against both *S. aureus* and *P*. *aeruginosa* (Kazemzadeh-Narbat et al., 2012). Additionally, they also validated the ideal cytotoxicity and antibacterial efficiency of AMP HHC36 *in vitro* through antibacterial experiments utilizing titanium surface nanotubes coated with a palmitoyl oleoyl phosphatidyl-choline (POPC) film after loading AMP HHC36 into a supersaturated calcium phosphate coating matrix for sustained drug release (Kazemzadeh-Narbat et al., 2013).

Yazici et al. (2019) aimed to enhance the delivery efficiency of AMPs to localized regions and consequently developed bifunctional peptides by fabricating titanium surface nanotubes through HA deposition. The dual functionality of these peptides was achieved by conjugating hydroxyapatite binding peptide-1 (HABP1) with AMP using a flexible linker. The resulting HABP1-AMP conjugate demonstrated favorable affinity for HA and exhibited high antibacterial activity against *S. mutans* (Gram-positive) and *E. coli* (Gram-negative), thus presenting a promising approach for targeted delivery of AMPs to specific areas.

### 3.2 Inorganic antibacterial substances loaded with CPCs

#### 3.2.1 TII-loaded CPCs

In the realm of biomaterial engineering stents, the integration of drug delivery functionality with therapeutic efficacy is a common strategy employed to augment stent performance. From a pharmaceutical standpoint, the manufacturing methodologies of stents must ensure compatibility with drug stability and sustained release profiles. In comparison to organic macromolecular drug molecules, TIIs exhibit several notable advantages, including reduced cost, superior stability, enhanced safety, and fewer constraints on the fabrication process of tissue-engineered scaffolds (Mouriño et al., 2012; Hoppe et al., 2013). Currently, in the ongoing research endeavors aimed at utilizing CPC as scaffolds for bone tissue engineering, researchers have selected specific metal ions as TIIs. These TIIs have been successfully validated for their notable efficacy in combating bacterial infections and enhancing the material matrix’s capacity to induce new bone formation (Gritsch et al., 2019; Feng et al., 2021).

Metals have been utilized as antibacterial agents and as materials for various biomedical research and applications (Zhong et al., 2023; Samuel et al., 2020; Makvandi et al., 2020). When CPC is employed as a drug delivery device for bone infections, a small quantity of ions within the calcium phosphate lattice can be substituted by other ions. Several studies have corroborated that the doping of metal ions, such as silver, copper, and zinc, into the bone cement matrix enables calcium phosphate to exhibit antibacterial properties (Kamphof et al., 2023).

Silver (Ag) is among the most extensively studied metal ions incorporated into calcium phosphate due to its remarkable antibacterial properties (Kamphof et al., 2023). Notably, silver nanoparticles (AgNPs) exhibit potent antibacterial activity by releasing silver ions that attach to the bacterial cell membrane, altering the lipid bilayer or surface charge state. This process generates reactive oxygen species (ROS) and free radicals, which damage organelles and biomolecules and regulate associated signal transduction pathways, ultimately leading to antibacterial efficacy (Zheng et al., 2018; Lee and Jun, 2019).

Choi et al. (2023) conducted a study evaluating the antibacterial effect of TTCP-dicalcium phosphate dihydrate (DCPD) cement impregnated with AgNPs. In a rat tibial infection model, local treatment with TTCP-DCPD cement containing varying concentrations of AgNPs was administered for either 3 or 12 weeks. The findings revealed a decrease in bacterial colony counts in the 12-week treatment group compared to the 3-week group. Furthermore, a trend towards a lower bacterial colony count was observed in groups treated with higher doses of AgNPs compared to those without AgNP impregnation. Separately, Köse et al. (2021) established a model of methicillin-induced osteomyelitis in the proximal tibia of New Zealand white rabbits. The animals were divided into three groups: the control group received vancomycin-impregnated PMMA bone cement beads, the experimental group received silver ion-doped calcium phosphate beads, and the negative control group received pure calcium phosphate beads. After 10 weeks, radiographic assessments demonstrated significant improvement in osteomyelitis in the experimental group compared to the control group. These results suggest that silver ion-doped calcium phosphate beads have the potential to stimulate bone tissue growth, resist infection, and ultimately treat experimental chronic osteomyelitis in animal models. However, it is crucial to acknowledge that the cytotoxicity of silver towards normal tissues and cells cannot be overlooked, posing a limitation to its widespread application in the treatment of bone infections (Mijnendonckx et al., 2013; Rizzello, 2014).

Copper (Cu), an essential trace element in the human body, possesses diverse biological functions, including broad-spectrum antibacterial effects, bone regeneration capabilities, and angiogenesis properties (Shen et al., 2021; Marziya et al., 2023; Holloway, 2019).

Foroutan et al. (2019) conducted a study to investigate the influence of Cu^2+^ release on the properties and underlying mechanisms of CPCs. They synthesized CPC containing varying concentrations of Cu^2+^ (0, 2, 4, and 6 mol%) through a room temperature precipitation reaction. *In vitro* experiments demonstrated that as the Cu content increased, the release of phosphorus and calcium decreased, while the release of Cu^2+^ escalated. Furthermore, an analysis of the antibacterial activity against *S. aureus* revealed that the antimicrobial potential of the bone cement enhanced with increasing Cu^2+^ concentration. Additionally, when human osteoblast-like osteosarcoma cells (Saos-2, HTB85) were inoculated onto the bone cement particles for a defined period, a significant increase in cell count was observed, thereby confirming its biocompatibility. This study underscores the dual benefits of copper-doped CPCs, which not only exhibit antimicrobial activity but also possess bone regeneration properties. Näf et al. (2024) developed bilayer nanocomposites consisting of PLGA and amorphous calcium phosphate, incorporating various copper nanoparticles, specifically copper oxide (CuONPs) and copper-doped tricalcium phosphate (CuTCPNPs). To assess the antimicrobial properties of these copper-containing materials, clinically isolated *S. aureus* and *S. epidermidis* were employed. Furthermore, the angiogenic potential of these nanocomposites was evaluated using the chick embryo chorioallantoic membrane (CAM) model. The findings revealed that both CuONPs and CuTCPNPs possess antimicrobial activities and serve as effective components for stimulating angiogenesis.

Moreover, numerous *in vitro* and *in vivo* experiments have demonstrated the antimicrobial properties of metals and their alloys, including magnesium, strontium, and zinc, when incorporated into CPCs against bone infections. Additionally, these metals have been shown to enhance the antimicrobial effectiveness of existing therapeutic agents (Daskalova et al., 2023; Sikder et al., 2020; Wang Y. et al., 2023; Dapporto et al., 2022; Shu et al., 2024; Wolf-Brandstetter et al., 2020). However, it is crucial to emphasize that the precise correlation between the antimicrobial activity and cytotoxicity of metal-doped CPCs remains to be elucidated in future investigations (Kamphof et al., 2023).

#### 3.2.2 Graphene-loaded CPCs

Graphene, an allotropic isomer of carbon, has been shown to enhance the mechanical properties of CPC matrices and improve their biocompatibility for bone repair and regeneration (Seonwoo et al., 2022; Ozder et al., 2023). Furthermore, studies have confirmed the antimicrobial efficacy of graphene against a broad range of bacteria, including both Gram-positive and Gram-negative strains (Akhavan et al., 2011; Gurunathan et al., 2012; He et al., 2015; Veerapandian et al., 2013).

Wu et al. (2020b) pioneered the preparation of an injectable CPC-CS-GO slurry through the doping of graphene oxide (GO) into CPC. This novel slurry exhibited a robust inhibitory effect against *S. aureus*, demonstrating an inhibition zone of 55.2 ± 2.5 mm, significantly surpassing that of the control CPC-CS (30.1 ± 2.0 mm) (p < 0.05). Furthermore, CPC-CS-GO displayed superior antibacterial activity against *S. aureus* biofilm *in vitro* (p > 0.05). Khosalim et al. (2022) synthesized biomaterials composed of HA, agarose, and GO. Incorporating 1.0wt% GO into these biomaterials resulted in a notable reduction of *S. aureus in vitro* colony-forming unit tests. Additionally, the excellent biocompatibility of these materials was confirmed through tests using mouse embryo osteoblast precursor cells (MC3T3-E1).

#### 3.2.3 Iodine-loaded CPCs

Iodine possesses a broad antimicrobial spectrum, demonstrating effectiveness against viruses, *Mycobacterium tuberculosis*, fungi, and bacteria (Tsuchiya et al., 2012). In a groundbreaking study, Morinaga et al. (2023) impregnated CPC with various concentrations of iodine to assess its *in vitro* antibacterial activity and *in vivo* biocompatibility. Their findings revealed that CPC containing 5% iodine retained a higher iodine content compared to other CPCs after 1 week of release. Furthermore, this CPC formulation exhibited antimicrobial efficacy against *S. aureus* and *E*. *coli* for up to 8 weeks, while maintaining a similar number of fibroblast colony formations as the control sample.

However, there are limited reports on the application of doping graphene or iodine into CPCs for the treatment of bone infections, and further exploration of these possibilities is warranted.

## 4 Multifunctional calcium phosphate-calcium sulfate complex bone cement for the treatment of bone infections

As a bone cement filler for the treatment of bone infections, it aims to promote functional recovery of the affected limb or area while ensuring the desired preventative and therapeutic outcomes. Consequently, orthopedic surgeons face stringent requirements in selecting an appropriate bone cement matrix. A crucial aspect of this selection process is ensuring that the degradation rate of the cement matrix aligns with the rate of new bone formation.

Although CPC possesses ideal biocompatibility and recapitulates the inorganic phase of human bone, exhibiting porosity that renders it a suitable carrier for the slow release of osteoconductive drugs (Ginebra et al., 2012; Vezenkova and Locs, 2022; Fang et al., 2006), it is recognized that its degradation rate is rather sluggish. Studies have demonstrated that the degradation and absorption of CPC *in vivo* requires over 20 weeks to complete (Russell et al., 2008; Bohner, 2000). Conversely, CSC, a frequently utilized bone defect filler in clinical practice, is associated with limitations such as its limited osteogenic potential, rapid drug elution rate (Mistry et al., 2016), and swift degradation rate *in vivo* (<12 weeks) (Zhao et al., 2020).

**FIGURE 3:**
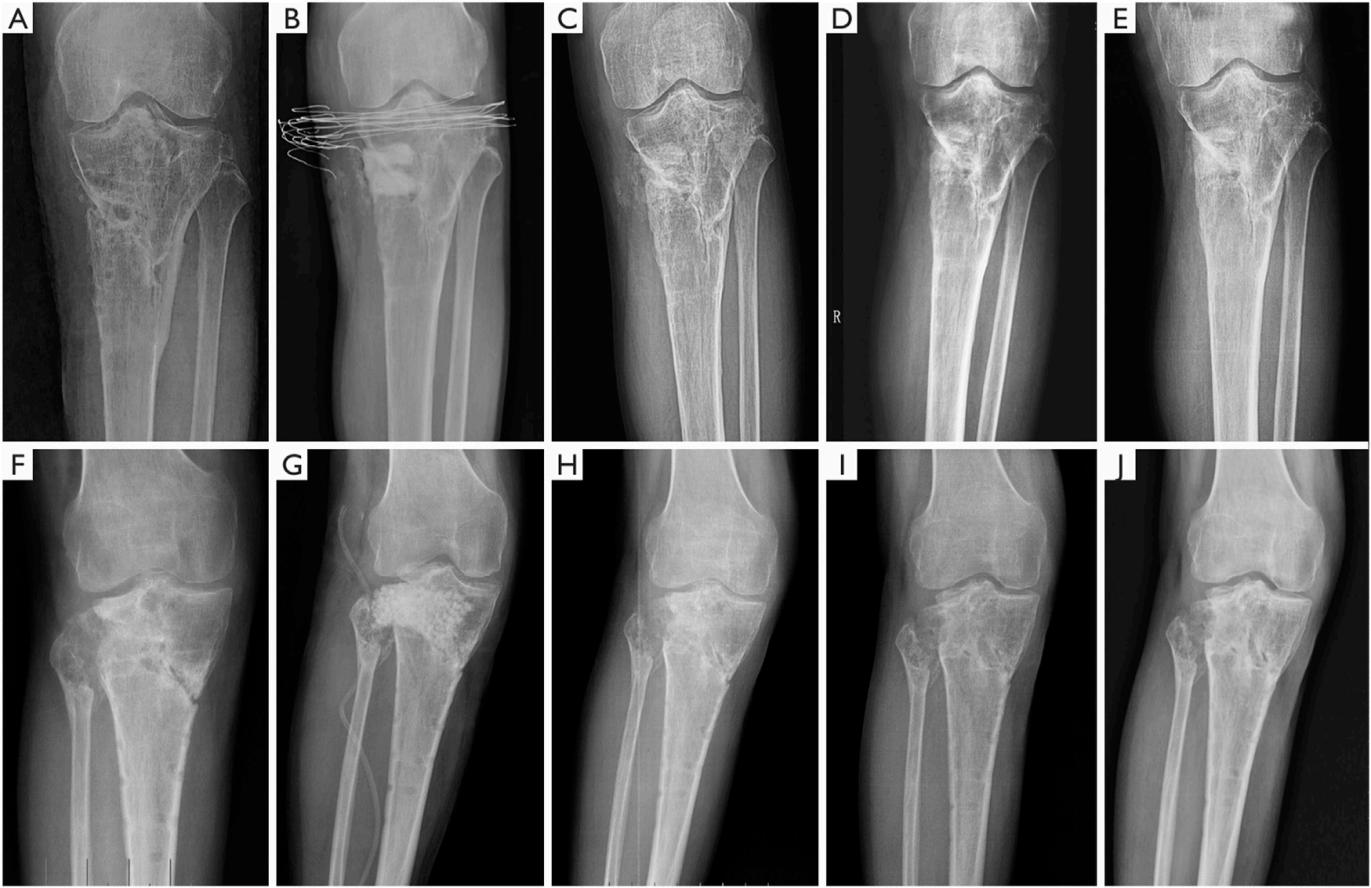
Typical radiographs of chronic osteomyelitis treated with CS/CP preoperatively **(A)**, immediately **(B)**, at 3 months **(C)**, at 6 months **(D)** and at 1 year **(E)** postoperatively. With the formation of new bone, the bone substitute composite was gradually absorbed and no new defects formed until the formation of new bone completed **(E)**. Typical radiographs of chronic osteomyelitis treated with CS preoperatively **(F)**, immediately **(G)**, at 3 months **(H)**, at 6 months **(I)** and at 1 year **(J)** postoperatively. Three months after the surgery, CS had been greatly absorbed. However, the new bone formation was slow and bone defect cavities still existed **(H, I, J)**. CS, calcium sulfate; CP, calcium phosphate (Zhao et al., 2020). Reprinted with permission from. Copyright 2020 Annals of Palliative Medicine.

To harmonize the resorption rate of the bone cement matrix with the rate of new bone formation, a multifunctional calcium phosphate-calcium sulfate complex bone cement was developed, exhibiting antimicrobial, osseointegrative, and degradable properties. Building upon the established potential of this cement as an effective antibiotic delivery system, as demonstrated by Mistry et al. (2016) through animal experiments, Zhao et al. (2020) reported the first clinical application of calcium phosphate (dicalcium phosphate (DCP) + β-TCP)-calcium sulfate complex bone cement impregnated with vancomycin in the treatment of chronic osteomyelitis caused by *S. aureus* infection. When compared to vancomycin-impregnated CSC used as a control, the composite bone cement exhibited superior new bone growth and a lower rate of infection recurrence (Table 2; Figure 3).

**TABLE 2:** Postoperative data of the two groups ( $\bar{x}$ ±s).

	Group A (CS/CP)	Group B (CS)	P value
Hospital stay (days), mean ± SD	35.05 ± 14.83	29.30 ± 7.12	0.156
Follow-up (weeks), mean ± SD	61.29 ± 33.75	61.29 ± 33.75	0.041*
Systemic antibiotic treatment duration (days), mean ± SD	36.33 ± 18,45	45.80 ± 8.92	0.064
Post-op ESR (mm/h), mean ± SD	9.10 ± 8.69	27.50 ± 26.24	0.056
Post-op serum hs-CRP (mg/L), mean ± SD	4.91 ± 7.78	15.65 ± 18.09	0.100
Post-op WBC (◊109), mean ± SD	6.65 ± 2.59	6.64 ± 1.48	0.984
Filling dose, mean ± SD	3.95 ± 1.56	4.49 ± 1.36	0.353
Defect length, mean ± SD	5.28 ± 0.98	5.66 ± 0.74	0.280
Defect width, mean ± SD	2.31 ± 0.50	2.28 ± 0.54	0.883
Microorganisms isolated (*Staph, E. cloacae, Bacillus subtilis*, negative)	14/1/1/5	5/1/0/4	0.643
Internal fixation	4/17	2/8	1.000
Complications	1/20	3/7	0.087
Recurrence	0/20	2/8	0.034*

*, P < 0.05. ESR, erythrocyte sedimentation rate; hs-CRP, high-sensitivity C-reactive protein; WBC, white blood cell count; CS, calcium sulfate; CP, calcium phosphate (Zhao et al., 2020). Reprinted with permission from. Copyright 2020 Annals of Palliative Medicine.

On the other hand, Mistry et al. (2022) employed biphasic calcium phosphate (BCP), consisting of HA blended with β-TCP and processed through foaming and high-temperature sintering, in the development of a calcium sulfate complex bone cement loaded with antibiotics (vancomycin + tobramycin). This formulation had been previously validated in animal experiments. It was utilized in the treatment of chronic osteomyelitis caused by MRSA infection, with gentamicin sulfate-loaded PMMA bone cement and intravenous vancomycin drip serving as control groups. Over time, clinical and radiological assessments demonstrated the superiority of the composite bone cement compared to the other two treatment modalities, evident in its antimicrobial efficacy, rapidity in sepsis control, and enhancement of new bone production.

Orthopaedic surgeons continue to grapple with the challenges of fracture-related infections (FRI) and periprosthetic infections (PJI). To address these issues, Freischmidt et al. (2020) introduced a novel, individualized surgical technique utilizing HA-calcium sulfate complexes impregnated with antibiotics (vancomycin or gentamicin sulphate). These composites were applied to intramedullary nails and plates, aiming to prevent biofilm formation and subsequent recurrence of bone or joint infections. The antimicrobial and osteoconductive properties of the composite bone cement coating the internal fixation devices yielded promising results in three reported cases of bone infection.

Recently, magnesium phosphate cement (MPC) has garnered significant attention as a novel bone repair material. Besides possessing advantages such as mechanical strength, biocompatibility, injectability, and modifiability, MPC exhibits superior bone conductivity and absorbability compared to CPC. These properties are particularly beneficial for bone tissue regeneration and repair. Furthermore, MPC offers a simpler operation and a degradation rate that is more in harmony with new bone formation (Tian et al., 2024; Ostrowski and Roy, 2016; Kaiser et al., 2022; Goldberg et al., 2020). Therefore, in comparison to calcium phosphate-calcium sulfate complex bone cement, MPC may exhibit comparable or even superior performance in exerting multiple effects simultaneously. However, this hypothesis necessitates further verification in subsequent studies.

## 5 Conclusion

Extensive research efforts have been directed towards addressing bone infections and the associated bone defects, focusing on osteointegration through the incorporation of antimicrobial drugs into CPCs or enhancing the physicochemical properties of CPCs. This review aims to present a comprehensive overview of various CPC-based antimicrobial agents, aimed at augmenting the antimicrobial efficacy and osteointegrative capabilities of bone cement fillers or coatings. While antibiotics remain the most extensively studied antimicrobial agents loaded into CPCs, the emergence of drug-resistant pathogens has garnered increasing attention. Additionally, several research teams have validated alternative antimicrobial agents, such as antimicrobial peptides and metal ions, through *in vitro* and animal experiments. The physicochemical properties of CPC play a pivotal role in both the antimicrobial activity of drugs loaded within the cement matrix and the stimulation of new bone formation. Notably, the interconnected pores inherent to calcium phosphate particles serve as reservoirs for antimicrobial agents. By encapsulating these pores with polymers, the localized and sustained release of these drugs can be modulated, thereby facilitating the expression of CPC’s osteoconductive and osteoinductive properties. Utilizing advanced techniques, antimicrobial drug-loaded calcium phosphates can be formulated as surface coatings on metallic endoprostheses (such as titanium alloys) or as slurries applied to endoprosthetic surfaces. This approach offers not only robust mechanical support but also localized antimicrobial and osseointegrative effects, thereby enhancing the overall therapeutic outcome.

However, a significant drawback of developing multifunctional CPC is the loading of diverse antimicrobial drugs. This loading process can compromise the physicochemical properties of the CPC matrix, thereby hindering the maximizaion of the antimicrobial efficacy of these drugs. Furthermore, the intricate nature of the antimicrobial CPCs fabrication process, coupled with the stringent regulations imposed by national legislatures and regulatory agencies regarding the approval of *in vivo* implants in the healthcare industry, has restricted the commercial viability of CPCs in the treatment of bone infections to a certain extent.

## 6 Future perspectives

In the future, multifunctional CPCs are poised to play a pivotal role in the prevention and treatment of bone infections. However, the sole reliance on calcium phosphate-loaded antimicrobial agents has proven insufficient in clinical practice. Therefore, it is imperative to integrate local drug delivery technology with antimicrobials to develop CPCs that possess optimal physicochemical properties, including antimicrobial activity, osseointegration promotion, and self-degradability. Additionally, it is crucial to mitigate cytotoxicity through the judicious combination of antimicrobial agents, such as bioactive metallic silver. Nonetheless, the pursuit of multifunctional modifications in bone cements often necessitates the utilization of multiple techniques, thereby increasing the overall cost and time required for the preparation of antimicrobial bone cements.

The development of anti-infective CPCs capable of specifically targeting pathogenic bacteria colonized within host cells, coupled with advanced targeting technologies, remains a pressing challenge for researchers. To address this challenge, future research should focus on modifying antimicrobial drugs while maintaining the biocompatibility and osseointegrative properties of CPCs, aiming to effectively treat chronic bone infections that persist due to intracellular bacterial colonization (Watkins and Unnikrishnan, 2020). Silver-copper-boron (AgCuB) nanoparticles (NPs), as prepared by Qadri et al. (2019), have demonstrated no harmful effects on host cells *in vitro* at concentrations ranging from 1 to 5 μg/g of CPC. *In vitro* experiments have shown that AgCuB NPs at concentrations of 1–5 μg/mL significantly reduce the internalization of *S. aureus* infection in osteoblasts in a dose-dependent manner, with a single treatment dose, without causing harm to host cells. To enhance the anti-cellular properties of AgCuB NPs, Abdulrehman et al. (2020) conjugated the cadherin-11 antibody (OBAb) to the original nanoparticles *in vitro*, resulting in AgCuB-OBAb NPs that could specifically target osteoblasts and subsequently exhibited remarkable antibacterial activity against intracellular *S. aureus*. Additionally, the use of liposomes for delivering antimicrobial drugs to host cells has been reported to effectively kill intracellular pathogens (Ferreira et al., 2021). The antimicrobial efficacy of liposomes can be further validated by utilizing targeting technology to identify specific markers on host cells and subsequently delivering the drugs to these cells efficiently.

The employment of bacteriophages (phages) in the combat against bone infections, notably those orchestrated by *S. aureus*, has garnered substantial attention. Phage therapy, in contrast to conventional antibiotic regimens, showcases remarkable benefits including pronounced heterogeneity and a diminished proclivity for eliciting bacterial resistance (Plumet et al., 2022; Kim et al., 2021). By efficiently lysing pathogenic bacteria and mitigating biofilm accumulation, phages assume an optimal stance in the prophylaxis and management of bone infections (Young et al., 2024). Notably, the therapeutic efficacy is further bolstered when phages are synergistically administered with antibiotics, heralding a novel, non-antibiotic approach to counteract drug-refractory bacterial pathogens implicated in bone infections (Moghadam et al., 2024; Bouchart et al., 2020). Innovatively, researchers have harnessed 3D-printed calcium phosphate bioceramics as targeted phage delivery vehicles, enabling direct infusion of phages to the infected site. This strategy not only mitigates systemic adverse effects but also augments therapeutic outcomes (Bouchart et al., 2020). Nevertheless, the clinical translation of phage-based interventions for bone infection prevention and treatment faces undeniable challenges, encompassing the safety profile, stability of the phages themselves, as well as regulatory hurdles that necessitate careful consideration and ongoing investigation.

Numerous studies have explored the application of photodynamic therapy (PDT) in the treatment of malignant tumors. Calcium phosphate, as a biomaterial with broad application potential in the biomedical field, has demonstrated anti-tumor effects when combined with PDT through the construction of bionic systems. This integration can involve strategies such as the combination of chemotherapy with PDT, pH responsiveness, and other mechanisms (Zhong et al., 2021; Nomoto et al., 2016; Liu et al., 2019). Recently, several studies have confirmed the beneficial effects of combining calcium phosphate with PDT in combating bacterial infections (Tan et al., 2024; Chen X. et al., 2024; Hu et al., 2022). In the pathological progression of infected bone defects, the advancement of infection serves as the primary obstacle to successful bone regeneration, thereby complicating treatment strategies. Consequently, the management of infectious bone defects necessitates a coordinated approach that addresses both anti-infection measures and the promotion of new bone formation. Tan et al. (2024) successfully incorporated 2D Ti_3_C_2_ MXene and berberine (BBR) into a 3D-printed calcium phosphate scaffold. Through both *in vitro* and *in vivo* experiments, the Ti_3_C_2_-BBR functionalized calcium phosphate scaffold exhibited remarkable antibacterial and osteogenic properties. The persistence of intracellular bacterial colonization in bone infections has posed a formidable challenge for an extended period. Traditional antibiotics targeting intracellular bacteria often struggle to achieve satisfactory anti-infective outcomes and may carry the risk of inducing bacterial resistance. Chen X. et al. (2024) introduced a novel photodynamic/photothermal calcium phosphate nanoparticle coated with mannose-based lipids (MAN-LCaP@Indocyanine green (ICG)) for the eradication of intracellular MRSA. Both *in vitro* and *in vivo* experiments demonstrated that MAN-LCaP, serving as a drug delivery vehicle, exhibited preferential uptake by macrophages and facilitated the transport of ICG to intracellular pathogens. MAN-LCaP@ICG offers a promising avenue for the clinical application in the treatment of anti-intracellular infections.

Furthermore, the immune response is frequently overlooked in traditional therapeutic approaches for bone infections. When CPC is implanted as an inlay material, it is recognized as a foreign substance by the immune system. Bone immunomodulation introduces a novel concept for antimicrobial bone cement, aiming to mitigate bacterial colonization (Chen et al., 2016). Additionally, the integration of immunomodulatory effects into biomaterials can mitigate the body’s excessive immune response locally, fostering the establishment of favorable osseointegration between the implanted material and surrounding bone tissues. Olechnowicz et al. (2018) demonstrated that zinc can reduce local tissue damage during repair by activating antioxidant enzymes, scavenging reactive oxygen species, and mitigating oxidative stress, thus promoting the progression of localized damage towards repair. Macrophages have emerged as crucial players in enhancing the osseointegration of CPCs (Li et al., 2020; Wu et al., 2022). It is crucial to highlight that, while pursuing the desired immunomodulatory effects, further research is imperative to elucidate strategies that safeguard the antimicrobial efficiency of CPCs and their physicochemical properties, vital for the formation of new bone, from being compromised.

## Glossary

CPC(s): Calcium phosphate cement(s)
TII(s): Therapeutic inorganic ion(s)
DFI(s): Diabetic foot infection(s)
*S. aureus*: *Staphylococcus aureus*
MRSA: Methicillin-resistant *Staphylococcus aureus*
OLCN: Osteocyte lacuno-canicular network
DFO: Diabetic foot osteomyelitis
PBP4: Penicillin-binding protein 4
SasC: Surface adhesin-S. aureussurface protein C
EPS: Extracellular polymeric substances
Agr: Accessory gene regulator
MSCRAMMs: Microbial surface components recognizing adhesive matrix molecules
SpA: Staphylococcal protein A
M-CSF: Macrophage colony-stimulating factor in osteoblasts
RANKL: Receptor activator of nuclear factor-κB ligand
DFU(s): Diabetic foot ulcer(s)
PMMA: Poly(methyl methacrylate)
CSC(s): Calcium sulfate cement(s)
TCP: Tricalcium phosphate
TTCP: Tetracalcium phosphate
OCP: Octacalcium phosphate
HA: Hydroxyapatite
CDHA: Calcium-deficient hydroxyapatite
MCPM: Monocalcium phosphate monohydrate
CS: Chitosan
HPMC: Hydroxypropyl methyl cellulose
PLGA: Poly (lactic-co-glycolic acid)
MIC: Minimum inhibitory concentration
AMX: Amoxicillin
PLA: Poly (lactic acid)
Ctr-mpHANCs: Citrate-modified mesoporous hydroxyapatite nanocarrier
CMC: Carboxymethyl cellulose
PS: Phosphoserine
S. epidermidis: *Staphylococcus* epidermidis
*E. coli*: *Escherichia coli*
*P. aeruginosa*: *Pseudomonas aeruginosa*
*K. pneumoniae*: *Klebsiella pneumoniae*
AMP(s): Antimicrobial peptide(s)
FDA: The U.S. Food and Drug Administration
POPC: Palmitoyl oleoyl phosphatidyl-choline
HABP1: Hydroxyapatite binding peptide-1
Ag: Silver
AgNP(s): Silver nanoparticle(s)
ROS: Reactive oxygen species
DCPD: Dicalcium phosphate dihydrate
Saos-2, HTB85: Human osteoblast-like osteosarcoma cells
CuONP(s): Copper oxide nanoparticle(s)
CuTCPNP(s): Copper-doped tricalcium phosphate
CAM: Chorioallantoic membrane
GO: Graphene oxide
MC3T3-E1: Mouse embryo osteoblast precursor cell(s)
DCP: Dicalcium phosphate
ESR: Erythrocyte sedimentation rate
hs-CRP: High-sensitivity C-reactive protein
WBC: White blood cell count
CS: Calcium sulfate
CP: Calcium phosphate
BCP: Biphasic calcium phosphate
FRI: Fracture-related infection(s)
PJI: Periprosthetic infection(s)
MPC(s): Magnesium phosphate cement(s)
AgCuB: Silver-copper-boron
NP(s): Nanoparticle(s)
OBAb: Cadherin-11 antibody
PDT: Photodynamic therapy
BBR: Berberine
MAN-LCaP: Calcium phosphate nanoparticle coated with mannose-based lipids
ICG: Indocyanine green
